# Beta-Carotene Supplementation for Sows: Reproductive Parameters and Productive Performance of Piglets

**DOI:** 10.3390/ani13233730

**Published:** 2023-12-01

**Authors:** Amanda Medeiros Araújo de Oliveira, Ednardo Rodrigues Freitas, Denise Batista Nogueira, Gabriel Gobira de Alcântara Araújo, Lucas Melo de Silva, Eduardo Afonso Frandi Butolo, Kassia Moreira Santos, Maurício Frias Prata, Claudia Cassimira da Silva Martins, Adsos Adami Passos, Carlos Lozano, Leonardo Augusto Fonseca Pascoal, Pedro Henrique Watanabe

**Affiliations:** 1 Departamento de Zootecnia, Universidade Federal do Ceará, Fortaleza 60020-181, Brazilednardo@ufc.br (E.R.F.);; 2 Departamento de Medicina Veterinária Preventiva e Saúde Animal, Universidade de São Paulo, São Paulo 05508-070, Brazil; 3 Topigs Norsvin, Curitiba 80420-210, Brazil; 4 Departamento de Ciências Animais, Universidade Federal Rural do Semi-Árido, Mossoró 59625-900, Brazil; lucasmeloufersa@gmail.com; 5 Integral Mix, Fortaleza 60831-745, Brazil; ebutolo16@gmail.com; 6 DSM Nutritional Product, São Paulo 05321-010, Brazil; 7 Departamento de Ciência Animal, Universidade Federal da Paraíba, Areia 58397-000, Brazil; leonardo@cchsa.ufpb.br

**Keywords:** antioxidant, carotenoid, hyper-prolificity, pigment, pro-vitamin, vitamin

## Abstract

**Simple Summary:**

Modern sows have an elevated metabolic rate, which can result in oxidative stress and impair reproductive performance. From this study, it is clear that dietary supplementation of beta-carotene can mitigate oxidative damage during lactation and increase piglet weight at weaning. When used from the beginning of gestation, beta-carotene supplementation also results in a greater number of weaned piglets and higher weaning weight.

**Abstract:**

The rapid fetal development and the increased demand for milk result in a catabolic state and oxidative stress in hyperprolific sows. Despite animal defense mechanisms, the dietary supplementation of antioxidants is being evaluated to reduce the impacts of excess free radicals. The aim of this study was to evaluate the short- and long-term effects of beta-carotene supplementation for sows on the reproductive response and performance of suckling piglets. A total of 120 sows were distributed in a 3 × 4 factorial arrangement of three supplementation levels (B0–no supplementation, B200–200 mg beta-carotene/day and B400–400 mg beta-carotene/day) and four groups of parity order (1st, 2nd, 3rd, above 4th). Beta-carotene supplementation during lactation resulted in a higher litter weight at weaning. A higher average weight of piglets and litter weight at birth were observed, as well as a greater number of piglets weaned and litter weight at weaning in sows supplemented with 400 mg of beta-carotene during gestation and lactation periods. Supplementation with 200 and 400 mg of beta-carotene resulted in a greater weight at weaning and daily weight gain in piglets. Daily supplementation with 400 mg of beta-carotene in the prepartum and lactation phases provides a greater litter weight at weaning and, when supplemented in the pre-gestation and gestation periods, results in a greater litter weight at birth and at weaning.

## 1. Introduction

In recent decades, the average size of sow litters has significantly increased due to genetic improvement, advances related to nutrition and advances applied to the management of animals in pig farms [1]. However, the rapid fetal development during pregnancy and the increased demand for milk during lactation result in fluctuations in the body condition of sows between reproductive cycles [2]. One of the main implications of the catabolic state during pregnancy and lactation in sows is the increase in oxidative stress [3,4], resulting from the high production of reactive oxygen species [5].

Although reactive oxygen species are produced by living organisms, as a result of normal cell metabolism, and are important markers of tissue remodeling, they are also related to a series of degenerative processes, due to their property of being or generating free radicals, the excess of which can cause lipid and protein oxidation and compromise the normality of endothelial cell function [4,6], as well as altering the formation of the placenta and fetus [7,8]. Although the body has enzymatic antioxidants as a defense mechanism to prevent damage caused by free radicals, the dietary supplementation of compounds with antioxidant action, such as beta-carotene, has been evaluated in mammals to reduce the negative impacts caused by excess circulating free radicals [9,10]. Beneficial fertility responses were obtained when beta-carotene was supplemented postpartum [11,12], and this is related to its conversion into vitamin A in the uterus and ovaries, being one of the possible mechanisms of action on reproductive efficiency [13].

Vitamin A is related to implantation and embryonic development, and also in the gene expression of steroidogenesis-related enzymes in the ovaries [14,15,16]. On the other hand, being the precursor of vitamin A, beta-carotene may have an antioxidant action, protecting cellular components from reactive oxygen species, and also acting on the mammary gland, where the increase in production and fat of the milk can be observed [11].

In light of the above, this study aimed to evaluate short- and long-term effects of beta-carotene supplementation for sows in different parity orders regarding reproductive performance.

## 2. Materials and Methods

The experimental procedures followed the protocols approved by the Committee of Ethics in the Use of Animals of the Federal University of Ceará, Brazil.

### 2.1. Animals, Treatments and Experimental Diets

A total of 120 sows (Topigs Norsvin^®^, Curitiba, Brazil), from the 1st- to 4th-parity orders, were distributed in a completely randomized design, in the 3 × 4 factorial arrangement, with three levels of beta-carotene supplementation and four groups of parity order. The beta-carotene supplementation levels were as follows: without beta-carotene supplementation; supplementation of 200 mg of beta-carotene per animal per day; supplementation of 400 mg of beta-carotene per animal per day. For the formation of parity groups, the sows were divided into 1st, 2nd, 3rd and above-4th parity orders (4th and 5th).

The experimental period lasted 196 days, comprised two reproductive cycles, started on the last gestational day and ended at the weaning of the subsequent cycle. The sows received supplementation for 30 days in the prepartum period, 21 days of lactation, five days of wean-to-estrus interval, 115 days of gestation and 21 days of lactation. Thus, in the subsequent period of gestation and lactation, they were grouped into 2nd, 3rd, 4th and above-5th parity orders.

All sows received diets and feeding management according to the phase (gestation, late-gestation and lactation). Diets were formulated to meet the minimum nutritional requirements in accordance with the recommendations by Topigs Norsvin^®^ (Table 1). Supplementation began at 85 days of gestation, with beta-carotene being introduced in the first feeding of the day throughout the experimental period. The addition of beta-carotene was carried out from a product with a concentration of 10% (ROVIMIX^®^ β-Carotene–DSM, São Paulo, Brazil). In order to guarantee beta-carotene intake, beta-carotene supplementation (200 and 400 mg of beta-carotene sow/day) was previously mixed with diet.

### 2.2. Animal Management

During late gestation (85 days), sows were fed 2.8 kg per day. At 110 days of gestation, sows were weighed, backfat thickness was measured (Lean-Meater, Renco Corporation, Golden Valley, MN, USA) and they were transferred to the farrowing house with individual farrowing crates containing feeders and drinkers for sows and piglets, in addition to a creep shelter with a heat source and a cooling system for the sow.

At lactation phase, sows were fed five times a day: the first at 6.00 am, the second at 8.00 am, the third at 10.00 am, the fourth at 2.00 pm and the last at 9.00 pm. The supplied amounts of diet and leftovers were weighed daily.

The sows were monitored during parturition and the total number of live piglets, stillborn, mummified and dead at birth were recorded. At birth, the piglets were submitted to the same postpartum managements, individually identified and weighed at birth. Cross-fostering was performed among piglets from sows of the supplementation group, until their third day of life, in order to maintain 13 to 14 piglets per sow. Feeding management during lactation consisted of providing 2 kg of feed on the first day after farrowing, with an addition of 1 kg of feed per day until reaching 7 kg of feed on the sixth day after farrowing. Piglets received pre-starter feed (Table 2) from the 7th day of life until weaning (21 days postpartum). At 21 days postpartum, sows were weighed, backfat thickness was measured (Lean-Meater, Renco Corporation, Golden Valley, MN, USA) and litters were weighed after weaning. Average daily milk production was estimated based on litter weight, litter weight gain and weaned piglets [17], not considering the weight gain attributed to feed intake by piglets.

Colostrum samples were collected on the day of birth, and milk was collected at 14 and 21 days of lactation, using 10 IU of injectable oxytocin in the auricular vein. Approximately 100 mL of milk was collected during manual milking of the functional teats of each sow, being homogenized and stored at a temperature of −20 °C for subsequent analysis. Colostrum and milk samples were analyzed for total solids, dry extract, protein, fat and lactose using the Lactoscan-Milk Analyzer, Milkotronic Ltd., Nova Zagora, Bulgaria.

After weaning, the sows were transferred to the gestation crates and, after estrus identification, inseminated again. During gestation, the sows received 1.7 kg of feed and, in the late-gestation period (85 days of gestation), 2.8 kg of feed was provided until transfer to the farrowing house.

At 110 days of gestation, the sows were weighed, the backfat thickness was measured (Lean-Meater) and they were transferred to maternity pen. The farrowings were followed up, using the same productive management procedures described above (counting of piglets born alive, stillborn, mummified and dead at birth). Cross-fostering was performed until the third day of life of piglets, in order to maintain 13 to 14 piglets per sow. The collection of colostrum and milk was performed on the day of birth, and milk was collected at 14 and 21 days of lactation for analysis (total solids, dry extract, protein, fat and lactose using the Lactoscan-Milk Analyzer device).

At 21 days postpartum, sows were weighed, backfat thickness was measured (Lean-Meater, Renco Corporation, Golden Valley, MN, USA) and litters were weighed after weaning. Average daily milk production was estimated based on litter weight, litter weight gain and weaned piglets [17], not considering the weight gain attributed to feed intake by piglets.

### 2.3. Statistical Analysis

Data were analyzed from sows and litters of 1st, 2nd, 3rd and above-4th parity orders, regarding beta-carotene supplementation for 51 days, and of 2nd, 3rd, 4th and above-5th parity orders for sows supplemented for 196 days, using the General Linear Models (GLM) procedure from the Statistical Analysis System (SAS University Edition, Cary, NC, USA). The model was

Ƴijk = µƳ + Ƭi + βj + Ƭβij + Ɛijk(1)

where Ƴijk is the value observed at the level of beta-carotene supplementation i (i = 0, 200 or 400 mg per day), at parity order j and on repeat k; µƳ is the population mean; Ƭi is the effect of the level of beta-carotene supplementation i; βj is the effect of parity order j; Ƭβij is the effect of the interaction of the level of beta-carotene supplementation i with the level of parity order j; Ɛijk is the experimental error associated with the observed Ƴijk value. The means were compared using the Tukey Test (*p* ≤ 0.05).

## 3. Results

During the first evaluated reproductive cycle, one sow from the B200 group and one sow (2nd parity order) from the B0 group were removed from this study due to reproductive problems. During the second evaluated reproductive cycle, seven sows from the B0 and B400 groups and eight sows from the B200 group were removed from the trial due to anestrus. 

Considering that the supply of beta-carotene was started in late gestation in the first evaluated reproductive cycle (Table 3), it was observed that dietary supplementation of beta-carotene during the late-gestation period had no effect on sow reproductive parameters. A parity order effect was observed on the total number of piglets, number of piglets born alive, litter weight at birth and at weaning, and average piglet weight at birth and at weaning. No effect of dietary beta-carotene supplementation was observed on stillborn, mummified and dead-at-birth piglets. In the second evaluated reproductive cycle (Table 4), the supplementation of 400 mg of beta-carotene for sows resulted in a higher litter weight at birth, in relation to those supplemented with 200 mg and those not supplemented; for the average piglet weight at birth, higher values were also observed for sows supplemented with 400 mg of beta-carotene in relation to those not supplemented, although it did not differ from the ones that received 200 mg of the additive. No effect of dietary beta-carotene supplementation was observed on stillborn, mummified and dead-at-birth piglets.

Regarding litter performance, it was observed in the first evaluated reproductive cycle that the supplementation of 400 mg of beta-carotene resulted in a greater weight of the litter at weaning, greater weight gain of the litter and higher estimated milk production. There was also an effect of parity order for sow weight at farrowing, sow weight at weaning, litter weight at weaning and average piglet weight at weaning. In the second reproductive cycle evaluated, a greater number of weaned piglets, litter weight at weaning and daily feed intake of sows supplemented with 400 mg of beta-carotene were observed in relation to sows from other treatments. For average piglet weight at weaning, litter and piglet weight gain at weaning and estimated milk production, higher values were observed for sows supplemented with 200 and 400 mg of beta-carotene compared to non-supplemented sows.

Regarding the composition of colostrum and milk (Table 5), there was no effect of supplementation on any of the parameters evaluated during the first evaluated reproductive cycle. For the subsequent reproductive cycle (Table 6), a higher total milk solids content was observed at 21 days of lactation in sows supplemented with 200 and 400 mg of beta-carotene compared to non-supplemented sows. For the first reproductive cycle, there was an effect of parity order for solids, fat and protein colostrum content and for milk composition at 14 days of lactation for solids, fat, protein and lactose content. For the second evaluated reproductive cycle, only the effect of the parity order on the colostrum solids content was observed.

## 4. Discussion

Sows supplemented in late gestation and in lactation produced heavier litters at weaning due to the greater daily gain and to superior milk production, suggesting better feed efficiency [18]. When supplementation was extended to the next reproductive cycle, the superiority of litter and piglets at weaning could also be attributed to the higher birth weight, with higher values being observed for piglets from sows supplemented with 400 mg of beta-carotene, in relation to those that did not receive it. The observed difference of 2.93 kg between litters of sows supplemented with 400 mg in relation to those not supplemented, corroborates the results obtained by Soares [19] who noted an increase in weight of 2.33 kg in the litter of sows with the parenteral supplementation of 180 mg of beta-carotene at weaning when compared to non-supplemented ones. In a previous study, Coffey and Britt [20] evaluated the intramuscular administration of 200 mg of beta-carotene for sows during the lactation period and found a difference of 0.80 kg. Elefson et al. [21] did not observe a gain in piglet performance when supplementing the maternal diet with beta-carotene from the 60th day of gestation. The studies’ results highlight the importance of supplementation timing. Beta-carotene supplied during late gestation and lactation was also used to combat oxidative stress in sows [11], and also attenuated the effects for piglets [9].

Krammer and Arurich [22] observed that the application of 70 mg of intramuscular beta-carotene on the day of weaning and after the last insemination resulted in a higher number of live born piglets, stating that the supply at the beginning of the gestation would be independent of its role as a precursor of vitamin A. In this sense, the dietary supplementation of beta-carotene from a previous lactation resulted in a numerical increase in live born piglets, which would be indicative of its positive effect on embryonic viability in sows [19], as follicular growth advances with the progression of lactation [23], which can define the estrus weaning interval. The duration of the weaning-to-estrus interval is associated with plasma changes in the levels of gonadotropins, steroids, inhibin, leptin, IGF-1 and insulin. In this sense, Madej et al. [24] stated that the effect of beta-carotene on reducing the level of cortisol could be related to the positive action of the additive on LH secretion and ovarian function, since high concentrations of cortisol promote an inappropriate ambient for follicular maturation due to the high concentration of free radicals that can cause apoptosis and death in various cell types [25], in addition to anomalies in fertilization and pre- and post-implantation embryonic development [26]. The retinal production of beta-carotene still plays a role in the development and maintenance of the endometrium and blastocyst implantation [14].

In a longer period of additive supply (196 days), sows fed with 400 mg of beta-carotene increased feed intake, maximizing the milk production and total solids content. According to Wang et al. [27], the health of the mammary gland, due to the action on the immune response generated in the milk-secreting cells of animals supplemented with beta-carotene, acts by reducing the incidence of symptomatic and asymptomatic pathologies that could decrease milk production. In this sense, the better performance on the litter may be related to the increase in the concentration of immunoglobulins in colostrum and milk, as observed by Chen et al. [28] when evaluating beta-carotene supplementation in sows. Furthermore, the effects of beta-carotene are closely related to its conversion into vitamin A in tissues, being an important regulator of cell differentiation, with a role in the morphogenesis of mammary glands and also protecting against oxidative stress [10]. Due to limited transplacental transfer, newborn mammals have low stores of vitamin A, significantly depending on the lactation period to acquire sufficient reserves and maintain adequate growth and development [29]. In this sense, dietary beta-carotene supplementation can have a positive impact on the quality of the female mammary system and the productive performance of the litter.

The greater quality of milk produced and factors related to the intestinal quality of piglets from the supplemented sows can result in a higher litter size and piglet weight at weaning, showing possible attenuate mortality. Li et al. [30] evaluated the oral supplementation of 40 mg and 80 mg of beta-carotene through gavage in suckling piglets, which resulted in better intestinal health in the treated animals, with less oxidative damage, less inflammation and a lower rate of enterocyte apoptosis.

Evaluating the effect of parity order, sow performance differed between reproductive cycles. Primiparous sows had a lower weight at farrowing and at weaning, showing a worse feed efficiency since they consumed the same amount of feed as the other sows. In these females, the litters and the piglets showed a smaller development than the other parity orders, although they had colostrum and milk with a higher content of total solids, fat and proteins. The results indicate that the higher the parity order, the greater the impact on mammary glands, agreeing with Yang et al. [31] who found a difference in the reproductive performance of sows according to parity order. In turn, improving the oxidative status in gilts to avoid second litter syndrome using antioxidant additives such as beta-carotene can be an important nutritional tool for hyperprolific sows [1]. Although beta-carotene requirement values for sows have not been established, the results presented in the present study indicate the benefits of its use aimed at improving the performance of modern replacement gilts.

## 5. Conclusions

Supplementation with 400 mg of beta-carotene in the prepartum and lactation phases provides greater litter weight at weaning and, when supplemented in the pre-gestation and gestation periods, results in greater litter weight at birth and at weaning.

## Figures and Tables

**Table 1:** Calculated and nutritional composition of experimental diets (B0) for sows in the gestation, late-gestation and lactation phases.

Ingredients (%)	Gestation	Late-Gestation	Lactation
Corn	78.398	72.085	54.223
Soybean meal	16.500	22.900	32.100
Wheat meal	-	-	2.000
Cellulose fiber ^1^	2.000	1.500	0.200
Sugar	-	-	3.000
Soy oil	-	-	3.800
Dicalcium phosphate	1.120	1.200	-
Limestone	0.700	0.8000	-
Salt	0.600	0.6000	-
Vitamin D (25OHD3) ^2^	0.025	0.025	0.025
Vitamin supplement ^3^	0.100	0.125	-
Mineral supplement ^4^	0.100	0.100	-
Vitamin and mineral premix ^5^	-	-	4.00
Mycotoxin adsorbent	0.100	0.100	0.100
DL-Methionine	-	0.065	0.047
Lysine HCl	0.054	0.086	0.087
L-Tryptophan	-	0.007	-
Guanidinoacetic acid ^6^ (60%)	-	0.100	0.100
Choline chloride (60%)	0.072	0.076	-
Biotin 2%	0.005	0.005	0.005
Phytase	0.005	0.005	
Prebiotic ^7^	0.100	0.100	0.100
Probiotic ^8^	0.006	0.006	0.006
Live yeast	0.100	0.100	0.100
Virginiamycin 50%	0.008	0.008	-
Cyromazine 25%	0.004	0.004	0.004
**Composition Calculated**			
ME (MJ/kg) ^9^	13.61	13.65	14.44
Crude protein (%)	14.01	16.70	19.81
Calcium (%)	0.78	0.86	0.95
Available phosphorus (%)	0.44	0.47	0.44
SID-Lysine (%)	0.60	0.77	0.97
SID-Methionine + cystine (%)	0.42	0.54	0.57
SID-Threonine (%)	0.43	0.52	0.63
SID-Tryptophan (%)	0.13	0.17	0.21
SID-Arginine (%)	0.80	1.07	1.31
SID-Valine (%)	0.58	0.69	0.82

^1^ OptiCell^®^. ^2^ Hy-D^®^. ^3^ Composition per kg of gestation diet: vitamin A (10,800 IU/kg), vitamin D (4560 IU/kg), vitamin E (72 IU/kg), vitamin K3 (2.7 mg/kg), vitamin B1 (1.8 mg/kg), vitamin B2 (5.4 mg/kg), vitamin B6 (3.15 mg/kg), vitamin B12 (0.036 mg/kg), niacin (40.5 mg/kg), pantothenic acid (13.5 mg/kg), folic acid (0.54 mg/kg), biotin (0.27 mg/kg). ^4^ Composition per kg of gestation diet: manganese (50 mg/kg), zinc (100 mg/kg), iron (100 mg/kg), copper (20 mg/kg), iodine (1 mg/kg), selenium (0.3 mg/kg). ^5^ Composition per kg of lactation diet: vitamin A (7000 IU/kg), vitamin D (4360 IU/kg), vitamin E (60 IU/kg), vitamin K3 (2 mg/kg), vitamin B1 (1.52 mg/kg), vitamin B2 (5 mg/kg), vitamin B6 (1 mg/kg), vitamin B12 (0.02 mg/kg), niacin (30 mg/kg), pantothenic acid (16 mg/kg), folic acid (0.6 mg/kg), biotin (0.4 mg/kg), manganese (40 mg/kg), zinc (100 mg/kg), iron (70 mg/kg), copper (60 mg/kg), iodine (1 mg/kg), selenium (0.3 mg/kg), L-carnitine (48.5 mg/kg), phytase (0.5 FYT/kg). ^6^ CreAMINO^®^. ^7^ *Bacillus subtilis, Bacillus licheniformes,* ^8^ *Saccharomyces cerevisiae.* ^9^ Metabolizable Energy.

**Table 2:** Composition of pre-starter diet.

Ingredients (%)	Pre-Starter
Corn	33.200
Soybean meal	12.800
Sugar	4.000
Protein source ^1^	5.000
Milk supplement ^2^	5.000
Pre-stater supplement ^3^	40.000

^1^ Quali Bios HD (Vaccinar, Belo Horizonte, Brazil): DL-methionine, salmon meal, poultry meal, L-lysine, L-threonine, yeast, blood plasma, calcium propionate, ethoxyquim. ^2^ Quali-leite (Vaccinar, Belo Horizonte, Brazil): sugar, silicon dioxide, dicalcium phosphate, lactose, micronized full fat soya. ^3^ Quali Start 400 (Vaccinar, Belo Horizonte, Brazil Brazil) composition per kg of diet: vitamin A (10,000 IU/kg), vitamin D (2000 IU/kg), vitamin E (50 IU/kg), vitamin K3 (2.0 mg/kg), vitamin B1 (1.5 mg/kg), vitamin B2 (5.0 mg/kg), vitamin B6 (2.0 mg/kg), vitamin B12 (0.02 mg/kg), niacin (30.0 mg/kg), pantothenic acid (15.0 mg/kg), folic acid (1.0 mg/kg), biotin (0.4 mg/kg), calcium (6.8 g/kg), phosphorus (4.4 g/kg), manganese (40 mg/kg), zinc (3200 mg/kg), iron (150 mg/kg), copper (200 mg/kg), iodine (1 mg/kg), selenium (0.4 mg/kg), choline (1000 mg/kg), colistin (40.0 mg/kg), lactose (90 g/kg), methionine (3.35 g/kg), lysine (7.86 g/kg), threonine (4.96 g/kg), tryptophan (1.3 g/kg).

**Table 3:** Reproductive parameters and litter performance of sows from different parity orders supplemented with beta-carotene in late-gestation and lactation (51 days).

Parameters	Beta-Carotene Supplementation (B)			Parity Order (PO)				SEM ^1^	*p* Value		
	0	200 mg	400 mg	1st	2nd	3rd	Above 4th		B	PO	B × PO
Sows, n	39	39	40	29	30	30	30				
Sow body weight, kg											
At farrowing	244.24	245.48	244.39	217.51c	237.03b	260.89a	262.46a	6.299	0.9906	<0.0001	0.4502
At weaning	202.70	202.39	203.29	168.29c	198.31b	217.59a	225.84a	7.026	0.8973	<0.0001	0.4931
Sow backfat thickness, mm											
At farrowing	13.40	13.76	13.86	13.55	14.15	14.08	13.67	0.113	0.7636	0.6355	0.6536
At weaning	10.90	11.07	11.19	10.55	11.15	11.43	10.47	0.138	0.9666	0.5633	0.5366
DFI ^2^, kg	5.83	5.63	5.57	5.99	5.66	5.50	5.59	0.192	0.3101	0.0876	0.5797
Litter size, n											
Total born	15.67	15.13	15.37	15.21ab	14.10b	16.60a	15.66ab	0.626	0.6055	0.0016	0.8993
Born alive	14.62	14.67	14.40	14.24ab	13.53b	15.77a	14.70ab	0.636	0.8838	0.0094	0.6529
Cross-fostering	13.47	13.77	13.60	14.14a	13.33b	15.60a	14.49ab	0.602	0.8563	0.0369	0.6236
At weaning	12.35	12.67	12.90	12.14	12.57	13.03	12.80	0.317	0.1597	0.0581	0.2938
Litter weight, kg											
At farrowing	19.82	20.30	20.40	18.62b	19.98ab	21.35a	20.67ab	0.879	0.7415	0.0288	0.2498
Cross-fostering	18.92	19.35	19.67	17.76b	17.66ab	20.95a	20.20ab	0.859	0.7236	0.0369	0.2266
At weaning	68.91b	72.34a	73.01a	61.86b	70.37a	73.66a	72.28a	2.503	0.0245	<0.0001	0.7378
Daily gain	2.38b	2.52ab	2.54a	2.10b	2.51a	2.51a	2.48a	0.115	0.0444	0.0002	0.7180
Average piglet weight, kg											
At farrowing	1.36	1.40	1.44	1.31b	1.49a	1.37ab	1.43ab	0.052	0.3046	0.0131	0.4828
At weaning	5.58	5.71	5.66	5.22b	5.83a	5.86a	5.68ab	0.171	0.7492	0.0028	0.7408
Daily gain	0.202	0.206	0.202	0.17b	0.20a	0.21a	0.20a	0.008	0.5103	0.0005	0.4010

^1^ Standard error of mean. ^2^ Daily feed intake. Means followed by different letters indicate significant differences between beta-carotene supplementation and parity order through Tukey test (5% of probability).

**Table 4:** Reproductive parameters and litter performance of sows from different parity orders supplemented with beta-carotene in late-gestation to lactation of ulterior reproductive cycle (196 days).

Parameters	Beta-Carotene Supplementation (B)			Parity Order (PO)				SEM ^1^	*p* Value		
	0	200 mg	400 mg	2nd	3rd	4th	Above 5th		B	PO	B × PO
Sows, n	32	31	32	24	24	24	23				
Sow body weight, kg											
At farrowing	271.10	263.13	268.79	240.26b	271.67a	279.31a	280.20a	1.855	0.0524	0.0001	0.2889
At weaning	226.07	221.46	227.24	201.20b	230.23a	229.77a	239.34a	2.174	0.4724	0.0001	0.0539
Sow backfat thickness, mm											
At farrowing	13.68	13.93	14.31	13.67	14.12	13.87	14.26	0.119	0.5663	0.4975	0.3623
At weaning	10.37	10.91	11.69	10.65	11.43	10.87	11.00	0.126	0.4937	0.4865	0.3266
DFI ^2^, kg	5.30b	5.89ab	6.17a	10.22	12.15	10.31	9.73	0.256	0.0412	0.3596	0.2678
Litter size, n											
Total born	15.00	15.02	15.49	14.57	16.03	15.37	14.70	0.231	0.5510	0.4059	0.2164
Born alive	13.65	13.81	14.50	13.67	14.68	14.17	13.43	0.245	0.5309	0.5965	0.2598
Cross-fostering	13.28	13.46	13.65	13.29	13.68	13.70	13.17	0.299	0.5349	0.4263	0.2677
At weaning	11.31b	12.25ab	12.56a	12.00	12.79	11.50	11.83	0.146	0.0340	0.1136	0.8787
Litter weight, kg											
At farrowing	19.17b	19.39b	22.10a	19.39	21.28	21.26	18.96	0.338	0.0427	0.3989	0.5562
Cross-fostering	18.94	19.38	20.05	19.14	20.52	20.69	18.70	0.3266	0.4563	0.6536	0.6533
At weaning	63.44c	71.66b	73.54a	58.97	72.38	65.11	61.59	1.248	0.0017	0.0628	0.4303
Daily gain	2.12b	2.49a	2.54a	1.90	2.46	2.11	2.04	0.053	0.0063	0.1640	0.2669
Average piglet weight, kg											
At farrowing	1.43b	1.44ab	1.55a	1.44	1.50	1.51	1.42	0.332	0.0162	0.7303	0.1647
At weaning	5.61b	5.85a	5.85a	5.24	5.73	5.52	5.34	0.074	0.0150	0.4793	0.2594
Daily gain	0.199b	0.210a	0.205a	0.18	0.19	0.19	0.18	0.002	0.0006	0.1854	0.7360

^1^ Standard error of mean. ^2^ Daily feed intake. Means followed by different letters indicate significant differences between beta-carotene supplementation and parity order through Tukey test (5% of probability).

**Table 5:** Estimated milk production, colostrum and milk composition at 14 and 21 days of lactation of sows from different parity orders supplemented with beta-carotene in late-gestation and lactation (51 days).

Parameters	Beta-Carotene Supplementation (B)			Parity Order (PO)				SEM ^1^	*p* Value		
	0	200 mg	400 mg	1st	2nd	3rd	Above 4th		B	PO	B × PO
EMP ^2^ (kg)	11.09c	12.45b	12.57a	10.04	12.20	13.47	12.36	0.658	0.0188	0.0536	0.3599
Colostrum, %											
Solids	25.02	25.55	25.78	26.49a	26.45a	24.95b	24.24b	0.319	0.5540	0.0259	0.9996
Fat	7.24	8.18	8.86	9.29a	8.58ab	8.62ab	7.53b	0.310	0.8366	0.0256	0.9640
Protein	16.20	16.16	16.34	16.65a	16.72a	16.00ab	15.28b	0.088	0.5252	0.0444	0.9964
Lactose	2.10	2.12	2.10	2.08	2.14	2.15	2.03	0.135	0.6513	0.8587	0.5838
Milk (14 days of lactation), %											
Solids	21.13	23.80	22.80	24.53a	22.78ab	22.61ab	20.24b	0.426	0.1003	0.0082	0.0723
Fat	8.41	8.58	9.15	8.46a	7.59b	8.76b	8.07b	0.336	0.2133	0.0193	0.0469
Protein	4.83b	5.33	5.23	5.57a	5.07ab	5.23ab	4.65b	0.112	0.1399	0.0019	0.1109
Lactose	4.85	5.02	4.97	5.16a	4.99ab	5.10ab	4.45b	0.063	0.2212	0.0011	0.1113
Milk (21 days of lactation), %											
Solids	18.79	18.89	18.67	17.67	19.25	18.22	20.24	0.394	0.9119	0.5511	0.4117
Fat	6.33	6.37	6.43	6.44	5.76	6.16	7.35	0.263	0.7251	0.0882	0.2410
Protein	5.63	5.58	5.66	5.58	5.54	5.46	6.00	0.110	0.9514	0.8313	0.5567
Lactose	5.53	5.40	5.51	5.68	5.44	5.34	5.43	0.090	0.9018	0.6286	0.0685

^1^ Standard error of mean. ^2^ Estimated milk production based on litter weight, litter weight gain and weaned piglets [17]. Means followed by different letters indicate significant differences between beta-carotene supplementation and parity order through Tukey test (5% of probability).

**Table 6:** Estimated milk production, colostrum and milk composition at 14 and 21 days of lactation of sows from different parity orders supplemented with beta-carotene in late-gestation to lactation of ulterior reproductive cycle (196 days).

Parameters	Beta-Carotene Supplementation (B)			Parity Order (PO)				SEM ^1^	*p* Value		
	0	200 mg	400 mg	2nd	3rd	4th	Above 5th		B	PO	B × PO
EMP ^2^ (kg)	8.64b	11.37a	11.92a	10.26	12.19	10.51	9.64	0.304	0.0009	0.1111	0.9576
Colostrum, %											
Solids	25.67	25.63	27.74	28.50a	27.29ab	26.25ab	23.17b	0.499	0.4973	0.0453	0.9483
Fat	7.21	7.9	7.97	7.34	8.16	8.25	7.26	0.494	0.8502	0.9485	0.4625
Protein	16.47	16.902	17.685	18.36	15.01	18.71	15.17	0.160	0.6667	0.1600	0.9434
Lactose	2.54	2.52	2.57	3.42	1.42	2.74	2.03	0.427	0.9509	0.4309	0.9329
Milk (14 days of lactation), %											
Solids	20.86	20.65	21.59	23.01	21.38	18.95	20.32	0.501	0.7941	0.2942	0.9955
Fat	8.2	8.92	8.9	8.82	7.52	9.44	8.52	0.112	0.7783	0.3113	0.8777
Protein	6.54	6.5	6.44	6.9	6.9	5.82	6.18	0.157	0.9979	0.3887	0.9902
Lactose	4.93	5.26	5.05	5.08	5.41	4.74	5.03	0.180	0.3812	0.3928	0.5061
Milk (21 days of lactation), %											
Solids	15.78b	18.30a	19.66a	19.19	16.20	17.78	17.65	0.500	0.0298	0.3982	0.3523
Fat	6.53	7.05	6.89	6.40	6.67	7.23	7.02	0.222	0.7334	0.8101	0.7603
Protein	5.33	5.78	5.90	5.66	5.80	5.58	5.64	0.153	0.5267	0.8839	0.5952
Lactose	4.94	5.63	5.15	5.25	5.57	4.52	5.37	0.296	0.7829	0.4668	0.7179

^1^ Standard error of mean. ^2^ Estimated milk production based on litter weight, litter weight gain and weaned piglets [17]. Means followed by different letters indicate significant differences between beta-carotene supplementation and parity order through Tukey test (5% of probability).

## Data Availability

The data supporting this study cannot be publicly shared, for privacy reasons, although may be shared upon reasonable request to the corresponding author if appropriate.
